# Assessment of nutrient adequacy in undergraduate students during the undertaking shipboard internship: using 12-day dietary recall through smartphone photography

**DOI:** 10.1186/s12889-023-17102-8

**Published:** 2023-11-29

**Authors:** Hyejin Chun, Jung-Heun Ha, Hyohyeon Im, Cho Young Jung, Miae Doo

**Affiliations:** 1 Department of Family Medicine, Ewha Womans University College of Medicine, Seoul, Korea; 2 Department of Food Science and Nutrition, Dankook University, Cheonan, Korea; 3 Research Center for Industrialization of Natural Neutralization, Dankook University, Yongin, Korea; 4 Department of Food and Nutrition, Kunsan National University, Gunsan, Korea Daehak-ro 558, Daehak-ro 558; 5 Division of Marine Industry-Transportation Science and Technology, Kunsan National University, Gunsan, Korea

**Keywords:** 24-hour dietary recall with smartphone photography, Nutritional assessment, Nutrient adequacy, Shipboard internships

## Abstract

**Background:**

This study aimed to investigated nutritional status and estimated the adequacy of dietary intake of university students during shipboard internships.

**Methods:**

In this cross- sectional study, data were collected from 25 students out of 42 participants who attended in the research information session in the department of maritime at a university located in Jeonbook, South Korea. The dietary intake data was obtained using the 12-day dietary recall through smartphone photography during the shipboard internships. The data on dietary intake were used to calculate acceptable macronutrient distribution ranges (AMDRs), frequency of inappropriate intake of the 2020 Dietary Reference Intakes for Koreans (KDRIs) as a reference, intake ratio to the nutrient adequacy ratio (NAR), mean adequacy ratio (MAR), and index of nutritional quality (INQ).

**Results:**

The average age of subjects was 21.68 years and average BMIs in men and women were 25.67 kg/m^2^ and 23.44kg/m^2^, respectively. The average energy of men and women was 2018.66 kcal and 1727.87 kcal, respectively. More than half of the subjects did not meet the inappropriate range of the AMDRs for carbohydrates and fat. The NAR of vitamin A, vitamin C, and calcium among all 10 nutrients tended to be lower in both men and women. The MAR were 0.71 and 0.769. On the other hand, in both men and women, vitamin C had the lowest INQ (0.5 and 0.39). For men, grains and potatoes were the major contributors to energy and carbohydrates, and calcium contributed in the order of meat, fish and eggs and vegetables and fruits. Although the highest contributors to energy and carbohydrates for women were grains and potatoes, the contributions from meat, fish, and eggs were similar, and the major contributors to calcium were vegetables and fruits.

**Conclusions:**

To improve the inadequate nutritional status of university students engaged in shipboard internships with the aim of pursuing careers as seafarers, there is a need to provide additional nutritional education tailored to their specific circumstances. Additionally, professional health guidance should be provided to maintain optimal nutritional status.

## Introduction

More than 2 million sailors between the 16th and 18th centuries died of scurvy, a vitamin C deficiency [1]. In 1800, it was recognized that citrus juice and fresh vegetables could cure scurvy [1]. However, they were difficult to store on board for a long period of time and easily spoiled before storage facilities, such as refrigerator-like storage, were developed. After that, vitamin C was discovered and isolated in the early 20C [1]. Although food storage facilities and techniques have been developed and vitamin C can be synthesized, even now, seafarers who stay aboard are limited in the quality and quantity of their diet by various factors [2]. Institutional food services are generally provided under the supervision of a professional dietitian, but most of them are provided only by cooks on board, and the cook decides what meals are served to seafarers on board [3]. Unfortunately, it is still difficult to store fresh ingredients until they reach the port during long voyages, so ingredients are stored in a frozen form to increase shelf life. In addition, there is a limit to the firepower that can be used for cooking on board 3. Therefore, for this reason, a previous study reported that the consumption of fresh fruits and vegetables on board is very low, and many animal product ingredients are used [4, 5].

An internship for undergraduate students is a structured and supervised work experience usually carried out during their academic studies [6, 7]. The purpose of organizing internships for undergraduates serves a dual role: first, to deepen their understanding of their chosen specialized fields, and second, to refine their vocational skills and professionalism by applying the academic theories they have acquired during their university tenure. For those aspiring to become seafarers, adhering to the Standards of Training, Certification, and Watchkeeping for Seafarers (STCW) convention introduces a unique requirement: university students are obligated to undergo a year-long, comprehensive practical shipboard experience [8]. This shipboard internship diverges from internships in other academic domains. Unlike internships in various fields, where students often participate for shorter durations and during specific time frames, maritime students engage in daily shipboard activities continuously for a 24/7 period, spanning weekends as well. Consequently, students undertaking shipboard internships frequently grapple with homesickness, feelings of isolation, malnutrition, and other challenges more intensely than their counterparts on land [9–11]. They are exposed to vibration and noise from ships, and they can experience stress and fatigue due to shipboard internships.

Although some studies have been conducted on the health and nutrition-related variables of seafarers, there is no study on the nutritional status of university students undertaking shipboard internships. Therefore, this study aimed to investigate the nutritional status of university students during shipboard internships using dietary intake with smartphone photography with and to determine the adequacy of daily energy and nutrients. Using smartphone photography, the 24-hour dietary recall method is a method that does not have to rely on the subject’s memory, so it is less burdensome and can reduce the error between actual intake and irradiation1 [12]. Furthermore, we identified the contributed ratio of dietary energy and various nutrients consumed from major food groups.

## Subjects and methods

### Recruitment of study subjects

This cross-sectional study was performed from June 2022 to February 2023 after holding two research information sessions (June 2022 and December 2022). The research information session was conducted for students in the department of maritime at a university located in Jeonbook, South Korea. The G-power software used for sample size was calculated for determine a large effect, 0.50 with a power of 0.8, using Wilcoxon-Mann-Whitney U test between means with a significance level of 0.05. of the 42 participants, a written informed consent form was obtained from 29 subjects who wanted to voluntarily participate. For the data analysis in this study, 25 students (17 men and 8 women) were included [13]. The Institutional Review Boards of Kunsan National University approved this study (IRB No. 1040117-202205-HR-008-02).

### Data collection

This study investigated data on general characteristics, anthropometric variables, and dietary intake. Anthropometric variables were collected immediately before taking a shipboard internship whereas general characteristics and dietary intake was collected during taking a shipboard internship. Anthropometric variables were height, weight, and waist circumference. Height and weight with wearing light clothes were measured using BSM 370 and InBody 570 (Biospace, Seoul, Korea), respectively. Waist circumference was measured between the upper hip bone and the top of the iliac crest with a tape measure.

General characteristics using an online survey were included in the data on age, gender, current smoking, alcohol consumption, subjective stress level, and physical activity. Current smoking and alcohol drinking were divided as “Yes (current smoking or drinking alcohol regularly)” or “No (never smoke/cessation of smoking or never drink alcohol)”. Subjective stress level was divided into high or low after assessment using a 4-point Likert scale [14]. Usual physical activity was assessed using the International Physical Activity Questionnaire (IPAQ) short form [15].

**Fig 1.**
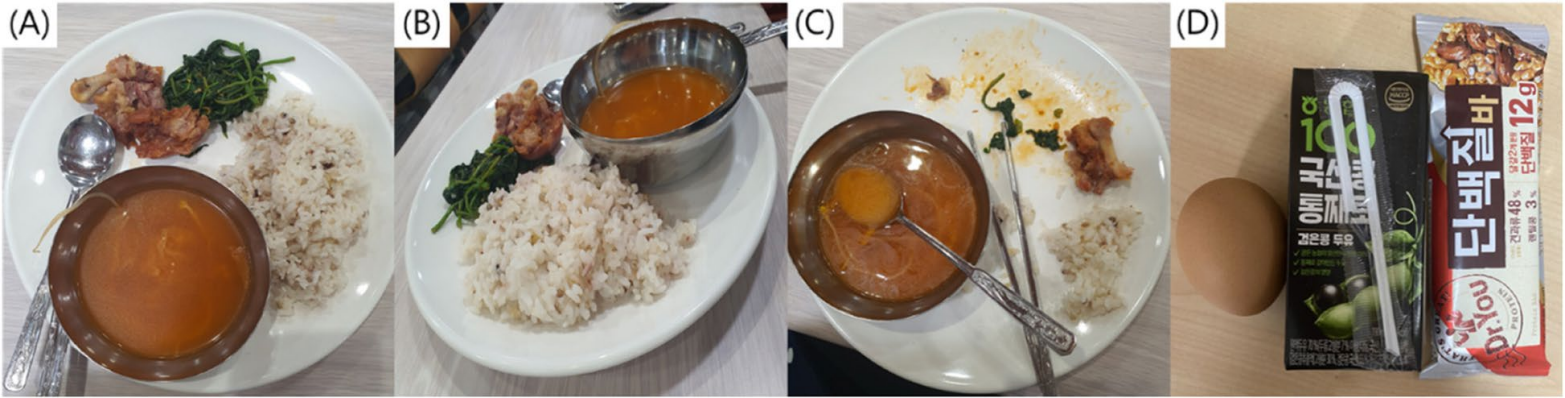
An example of the dietary intake survey using smartphone photography. **A**, Before the meal from above; **B**, before the meal from the side; **C**, after the meal; **D**, snack

Dietary intake was investigated for a total of 12 days, 3 times a week (2 days on weekdays and 1 day on weekends) during 4 weeks of shipboard internships. Consumed subjects’ food items and amounts were photographed with their smartphones immediately before and after the meal and snacks and then sent individually to the trained dietitians, except in cases where internet access is not available during the training ship (Fig 1). After the internship was completed, all dietary intake survey using smartphone photography were reviewed to check for inappropriate or missing records. To accurately examine usual dietary intake, the menu, plates, and bowls provided on the training ship were receive. The dietary intake using photography was analyzed weight portion by trained dietitian.

### Assessment of food groups and nutrients

The data on dietary intake were analyzed using CAN Pro 5.0 (Computer-aided nutritional analysis program for professionals, Seoul, Korea), a nutrient database developed by the Korean Nutrition Society, and then converted to nutrient consumption. Assessment of dietary intake was used as the average nutrient intake of 12-day dietary records. Average daily nutrient and food group intake, acceptable macronutrient distribution ranges (AMDRs), frequency of inappropriate intake of the 2020 Dietary Reference Intakes for Koreans (KDRIs) as a reference, intake ratio to the nutrient adequacy ratio (NAR), mean adequacy ratio (MAR), and index of nutritional quality (INQ) were calculated [16–18]. AMDRs were calculated as the percentage of energy intake from carbohydrates, protein, and fat, which are macronutrients, to total energy intake. The prevalence of subjects with inadequate intake was calculated for energy, protein, dietary fiber, cholesterol, and 11 micronutrients (vitamin A, thiamine, riboflavin, niacin, folic acid, vitamin C, calcium, phosphorus, iron, sodium, and potassium). The intake of nutrients such as protein, calcium, phosphorous, iron, vitamin A, thiamine, riboflavin, niacin, folic acid, and vitamin C was calculated as a ratio to the estimated average requirement (EAR), energy intake was calculated as a ratio to the estimated energy requirement (EER), sodium and cholesterol intake was calculated as a ratio to the recommended value and chronic disease risk reduction intake (CDRR), and dietary fiber and potassium intake was calculated as a ratio to the adequate intake (AI) among the 2020 KDRIs for an individual’s gender and age. To examine the adequacy of an individual’s nutrient intake, NAR was calculated for protein, calcium, phosphorous, iron, vitamin A, thiamine, riboflavin, niacin, folic acid, and vitamin C. NAR values of each nutrient were 1 or more, which was regarded as 1, and the individual’s nutrient intake was assessed for adequacy. MAR was calculated as the average NAR of 10 nutrients to measure the overall quality of dietary intake. INQ was calculated as the ratio of nutrient intake per 1000 kcal to the RDA of that nutrient per 1000 kcal to assess the nutritional balance of the diet.

$$NAR= \frac{Dalily\, specific \,nutrient\, intake}{Reference\, intake\,of\, specific\, \,nutrient}$$

$$MAR= \frac{\sum NAR}{Number\, of\, nutrients}$$

$$INQ=\frac{Amount\, of\, specific\, nutrient\, in\,1000kcal\, of\, diet\, intake}{Reference\, intake\, of \,specific\, nutrient\, per\, 1000kcal}$$

Food items were categorized into five food groups (grains and potatoes; meat, fish, and eggs; legumes and nuts; vegetables and fruits; and dairy) based on common food groups according to the Korean Nutrient Database.

### Statistical analyses

Before the data were analyzed, all continuous variables were assessed for normal distributions. Differences by gender were determined by Fisher's exact test or the Mann‒Whitney U test. A *p* value of <0.05 was considered significant. All statistical analyses were carried out using SPSS (version 27.0; IBM Corp., Armonk, NY, USA) software for Windows.

## Results

### General characteristics

The general characteristics of the study subjects are presented in Table 1. The average age was 21.68 years (SD = 2.04), and 68.00% of the total subjects were men. Current smokers and drinkers among men subjects were 4.7 and 7.1 times more likely than those among women subjects, respectively (*p*<0.05 for current smoking and *p*<0.001 for current alcohol consumption). Women subjects experienced high stress status and less physical activity than men subjects. The average BMIs in men and women were 25.67 kg/m^2^ and 23.44kg/m^2^, respectively.

**Table 1:** Characteristics of the study subjects before taking a shipboard internship

	**Men (*n*=17)**	**Women (*n*=8)**	***P* value**
Age, years	22.29±2.17	20.38±.074	0.011
Current smoking, smoker	58.82	12.50	0.042
Current alcohol consumption, drinker	88.24	12.50	0.001
Subjective Stress status, high	41.18	50.00	1.000
Physical activity, METS	3467.12±3118.11	1833.38±2317.67	0.262
Heigh, cm	173.82±11.26	163.18±7.02	0.003
Weight, kg	79.23±13.33	62.54±10.31	0.005
BMI, kg/m^2^	25.67±4.62	23.44±3.08	0.344
Waist circumference, cm	86.06±9.88	70.25±4.76	<0.001

Values are presented as the mean±SD or %. *P* value was determined using Fisher's exact test or Mann‒Whitney U test.

### Dietary nutrients intake and number of inadequate intake subjects

The intake of dietary energy and nutrients and the number of inadequate intake subjects are presented in Table 2. The average dietary energy of men and women was 2018.66kcal and 1727.85kcal, respectively, and the inadequate subjects based on the EER accounted for 58.82% and 75.00%, respectively. As an absolute quantity, dietary macronutrient intake was adequate with respect to the 2020 KDRIs in both men and women. However, half of the subjects did not meet the appropriate range of the AMDRs, except for protein, which was the relative proportion of macronutrients from energy intake. More than approximately seventy percent of men subjects had an inappropriate range of the AMDRs for carbohydrates (76.47%) and fat (70.59%); on the other hand, 62.50% and 50.00% of women subjects had an inappropriate range of the AMDRS for carbohydrates and fat, respectively. The average dietary fiber intake of men and women was 14.75 mg and 13.51g, respectively, and all subjects consumed less than AI. The average cholesterol intake of men and women was 399.52 mg and 293.31 mg, respectively, and the percentage of subjects who consumed more than the recommended intake of 300 mg based on the 2020 KDRIs was 76.47% for men and 37.50% for women, which was approximately twice as high for men as for women.

**Table 2:** Dietary nutrient intake and number of inadequate intake subjects

	Men (*n*=17)		Women (*n*=8)		*P* Value^c^	*P* value^d^
	Intake^a^	Inadequate intake^b^	Intake^a^	Inadequate intake^b^		
Energy, kcal	2018.66±369.57	58.82	1727.85±338.78	75.0	0.075	0.072
Carbohydrate, g	260.06±45.267		230.07±47.35		0.215	
Protein, g	82.79±17.86		70.47±16.00		0.124	
Fat, g	63.75±15.62		54.27±11.17		0.097	
Dietary fiber, g	14.75±4.35	100.00	13.51±3.90	100.00	0.588	-
Cholesterol, mg	399.52±137.18	76.47	293.31±112.77	37.50	0.124	0.087
Vitamin A, ugRAE	319.02±80.27	100.00	297.60±96.30	100.00	0.932	-
Thiamine, mg	1.91±0.48	5.88	1.62±0.24	0.00	0.066	1.000
Riboflavin, mg	1.36±0.34	35.29	1.17±0.30	37.50	0.288	1.000
Niacin, mg	14.65±3.63	23.53	12.70±3.56	37.50	0.374	0.640
Folate, ug	367.30±118.27	35.29	310.57±100.13	62.50	0.288	0.389
Vitamin C, mg	39.98±15.80	100.00	34.73±18.33	100.00	0.344	-
Calcium, mg	327.39±87.73	100.00	334.67±93.19	100.00	1.000	-
Phosphorus, mg	1050.49±233.06		887.35±221.16	12.50	0.097	0320
Iron, mg	16.49±2.84		15.04±2.79		0.175	
Sodium, mg	3386.62±688.73	94.11	2945.26±720.28	87.50	0.140	1.000
Potassium, mg	1924.71±438.88	100.00	1734.80±440.04	100.0	0.374	-
Energy distribution						
Carbohydrate, %	51.87±4.64	76.47	53.34±3.46	62.50	0.374	0.640
Protein, %	16.36±1.42		16.25±1.39		0.475	
Fat, %	31.77±4.03	70.59	30.41±2.69	50.00	0.440	0.394

^a^ Average intake of dietary energy, nutrients, and energy distribution of macronutrients; values are presented as the mean±SD

^b^ Prevalence of inadequate intake reference to KDRIs. Values are presented as %. KDRIs: 2020 Dietary Reference Intakes for Koreans; Energy was used < 75% or ≥ 125% for estimated average requirement (EER); dietary fiber, potassium were used below for Adequate intake (AI); cholesterol and sodium were used recommended value and chronic disease risk reduction intake (CDRR); and other nutrients were used below for estimated average requirement (EAR); Energy distribution were used < or ≥ for acceptable macronutrient distribution ranges (55-65% for carbohydrate; 7-20% for protein; 15-30% for fat)

^c^ *P* value for nutrient intake was determined using Mann‒Whitney U test

^d^ *P* value for inadequate intake was determined using Fisher's exact test

Among vitamins and minerals, all subjects consumed less than EAR for vitamin A, vitamin C and calcium, indicating that men consumed 319.02 µg RAE, 39.98 mg, and 327.39 mg and women consumed 297.60 µg RAE, 34.73 mg, and 334.67 mg, respectively. The average folate intake for men and women was similar at 367.30 mg and 310.57 mg, respectively; however, 62.50% of women and 35.29% of men had folate intake less than the EAR. As a reference for the CDRR for sodium, most subjects (94.11% for men and 87.50% for women) consumed 3386.62 mg and 2945.26 mg in excess.

### Nutritional adequacy assessment

NRA, MAR, and INQ are presented in Table 3. Among all micronutrients, the NAR of vitamin A, vitamin C, and calcium was low: 0.40 and 0.46 for vitamin A, 0.40 and 0.35 for vitamin C, and 0.41 and 0.48 for calcium for men and women, respectively. The MARs of all 10 nutrients were 7.71 and 7.69 for men and women, respectively. On the other hand, INQ, which assesses the adequacy of diet considering energy intake, showed a different trend from the nutrients of NAR. In both men and women, vitamin C had the lowest INQ (0.50 and 0.39) among all 10 nutrients. However, the INQ of iron for men was highest at 2.20, followed by thiamine (2.03) and phosphorus (1.92). Thiamine for women was highest at 1.72 among all 10 nutrients.

**Table 3:** Nutrient adequacy ratio (NAR) and index of nutritional quality (INQ)

	NAR			INQ		
	Men (*n*=17)	Women (*n*=8)	*P* value	Men (*n*=17)	Women (*n*=8)	*P* value
Protein	0.98±0.06	0.98±0.05	0.977	1.64±0.14	1.48±0.12	0.009
Vitamin A	0.40±0.10	0.46±0.15	0.440	0.52±0.11	0.52±0.10	0.887
Thiamine	0.98±0.06	1.00±0.00	0.669	2.03±0.24	1.72±0.13	0.001
Riboflavin	0.86±0.18	0.89±0.16	0.475	1.16±0.16	1.12±0.12	0.711
Niacin	0.87±0.16	0.86±0.20	0.887	1.17±0.14	1.04±0.15	0.086
Folic acid	0.84±0.21	0.75±0.21	0.374	1.16±0.24	0.88±0.13	0.009
Vitamin C	0.40±0.16	0.35±0.18	0.344	0.50±0.14	0.39±0.14	0.057
Calcium	0.41±0.11	0.48±0.13	0.315	0.53±0.12	0.55±0.08	0.440
Phosphorus	1.00±0.01	0.98±0.06	0.588	1.92±0.14	1.46±0.10	<0.001
Iron	1.00±0.01	0.96±0.06	0.194	2.20±0.61	1.26±0.18	<0.001
MAR	7.71±0.92	7.69±1.08	0.932	-	-	

Values are presented as the mean±SD. *p* value was determined using Mann‒Whitney U test. *NAR* Nutrient adequacy ratio, *MAR* Mean adequacy ratio, *INQ* Index of nutritional quality

### Contribution of five common food groups to daily nutrients intake

The contributions of the 5 common food groups to daily energy and nutrients consumed are presented in Table 4. The intake of total daily energy, carbohydrates, and calcium obtained from the food groups showed differences according to gender. For men, grains and potatoes accounted for the highest contributions of 59.3% and 89.7% of total energy and carbohydrates, and calcium intake was contributed in the order of meat, fish, and eggs (39.8%) and vegetables and fruits (32.4%). Although the highest contribution of total energy and carbohydrates for women was grains and potatoes (46.9% for total energy and 54.9% for carbohydrates), the contributions from meat, fish, and eggs were similar (45.2% for total energy and 38.1% for carbohydrates). Interestingly, 39.8% of vegetables and fruits as a source of calcium consumed by women were the highest, followed by 27.5% of meat, fish, and eggs.

**Table 4:** Contribution of food groups to total daily energy and nutrient intakes

	Men (*n*=17)					Women (*n*=8)				
	Grains and potatoes	Meat, fish, and eggs	Legumes and nuts	Vegetables and fruits	Dairy	Grains and potatoes	Meat, fish, and eggs	Legumes and nuts	Vegetables and fruits	Dairy
Energy	59.30	33.49	1.33	4.42	1.46	46.88	45.18	1.55	4.70	1.69
Carbohydrate	89.68	2.58	0.26	6.46	1.02	54.90	38.10	0.27	5.60	1.14
Protein	24.54	65.89	2.33	5.67	1.57	28.68	57.67	3.68	8.05	1.92
Fat	9.43	83.28	3.31	1.32	2.66	15.12	70.58	7.04	2.20	5.07
Dietary fiber	34.13	11.22	6.28	48.09	0.28	26.05	22.04	4.93	46.74	0.24
Vitamin A	15.62	28.84	0.01	51.09	4.44	14.19	23.18	0.00	57.41	5.22
Thiamine	27.62	55.55	1.58	14.27	0.98	29.30	49.20	2.34	17.81	1.35
Riboflavin	13.95	67.02	0.94	14.42	3.67	18.62	53.92	1.31	20.34	5.80
Niacin	29.36	59.48	0.81	9.90	0.45	31.01	53.33	1.19	13.88	0.59
Folate	20.97	24.29	1.11	52.47	1.16	19.38	24.10	1.45	53.70	1.37
Vitamin C	14.35	2.32	0.11	81.03	2.18	10.07	9.15	0.08	76.37	4.34
Calcium	10.34	39.82	4.15	32.36	13.33	11.38	27.17	6.50	39.77	15.18
Phosphorus	25.79	57.21	3.13	9.98	3.88	28.43	49.89	4.46	12.74	4.48
Iron	30.73	45.01	8.69	15.20	0.38	26.86	39.52	15.36	17.79	0.47

Values are presented as %.

## Discussion

This study stands out due to its objective of assessing nutritional status and estimating the adequacy of dietary intake among university students engaged in shipboard internships with the aim of pursuing careers as seafarers. Indeed, it investigates the contributed ratio of nutrients consumed from major food groups. Upon analyzing the collected 24-hour dietary recall data, a notable pattern emerged, indicating significant inadequacy in the consumption of both macronutrients and micronutrients among the internship participants. These participants demonstrated energy intake levels lower than the guidance provided by the Korean Dietary Reference Intake (KDRI). More than half of the students tended to consume less than 55-65% of the AMDRs optimal value for carbohydrates and more than 15~30% of those for fats. Of the 10 micronutrients, in both men and women, the NAR and INQ of vitamin C were the lowest. Interestingly, the source of carbohydrate was grains and potatoes for men, whereas meat, fish, and eggs contributed in similar proportions to women, as well as grains and potatoes. In addition, as a major source of calcium, vegetables and fruits were predominant in both men and women.

### Comparison of general characteristics with same age groups

When compared with the Korean National Health and Nutrition Examination Survey (KNHNES) data 2021 of the same age group [19], the smoking status of men students undertaking shipboard internships practice were twice as high as 29.38% of men of the those in KNHNES, however, current alcohol drinking status of women students undertaking shipboard internships were 4.56 times lower times than that of 57.02% of women of the those in KNHNES. Subjective stress level was found to be higher in both men and women than in the same age group from KNHNES 2021. Although the measurement tools were different, it was confirmed that the physical activity level was about twice as high for men and women students undertaking shipboard internships than those in the same age group from KNHNES 2021.

### Status of dietary energy intake and acceptable macronutrient distribution ranges

Our study observed the dietary status of both men and women participants, revealing that they consumed significantly less energy than the recommended EER of 2,600 kcal for men and 2,100 kcal for women, respectively, which is in line with the KDRI [15]. The EER is not an absolute value but could be recommended differently depending on the individual’s height, body weight, and physical activity level. It was confirmed that the physical activity level was about twice as high for men and women students undertaking shipboard internships than for those in the same age group from KNHNES 2021 [15]. Given that the subjects were highly physically active, it is likely that their energy intake was insufficient. The average consumed their energy from carbohydrates for men and women was found to be both falling below the lower limit of the AMDR of 55% for carbohydrates recommended for Koreans. Conversely, the average energy intake from fat for men and women was found to exceed the upper limit of the 30% fat AMDR for Koreans. Over the last 50 years in South Korea, the consumption of animal-source foods has continued to rise, while carbohydrates have decreased [20, 21], coinciding with the growing popularity of low-carbohydrate, high-fat diets for weight loss among Korean young adults [22]. Notably, a study called the Atherosclerosis Risk in Communities (ARIC) study reported that both very high and very low carbohydrate intake is associated with higher mortality and that an intake of 50-55% of carbohydrates is linked to the lowest risk of mortality [23]. Therefore, education on appropriate AMDRs for macronutrients is needed, and appropriate diets need to be provided.

### Inappropriate intake of the 2020 Dietary Reference Intakes for Koreans as a reference

The consumption of dietary fiber and cholesterol is closely associated with variations in carbohydrate intake, with lower carbohydrate consumption linked to higher cholesterol intake. In our study, all participants fell short of meeting the AI level for dietary fiber. While the functional benefits of dietary fiber vary based on factors such as solubility, viscosity, and fermentability, it is well established that fiber plays a crucial role in preventing metabolic complications such as insulin resistance, cardiovascular disease risk, gut abnormalities, and specific types of cancer [24, 25]. On a different note, a significant proportion of the subjects exceeded the recommended 300 mg of dietary cholesterol according to the 2020 KDRIs [15]. A report from the 2015 Dietary Guidelines Advisory Committee indicated that there exists a vague correlation between dietary cholesterol intake and circulating cholesterol levels, and dietary cholesterol might not be a primary contributor to dyslipidemia [26]. The report suggested reducing cholesterol consumption in metabolically complex groups, such as diabetic patients who are at a higher risk of developing cardiovascular disease [26]. To mitigate dietary cholesterol intake, the report recommended adopting a dietary pattern rich in vegetables, fruits, whole grains, lean meats, poultry, fish, nuts, fat-free or low-fat dairy products, and vegetable oils [27]. Notably, foods that are effective in lowering cholesterol are often intertwined with those high in dietary fiber. Therefore, the observed pattern of lower fiber intake but higher cholesterol consumption among internship students could largely be attributed to their food choices. Conversely, most subjects consumed more sodium than the CDRR threshold [17]. A high intake of sodium is associated with an increased risk of various chronic diseases, such as hypertension, stroke, and cardiovascular disease. A recent study reported that seafarers are at an increased risk of cardiovascular disease due to common unhealthy eating patterns, smoking, hyperlipidemia, and obesity [28]. Considering our results, students undertaking shipboard internships need to focus on healthy eating management to prevent chronic diseases and become healthy seafarers.

### Nutritional adequacy

The deficiency in dietary intake among students undergoing internships extends beyond just macronutrients; it encompasses micronutrients as well. Among the subjects, 29.4% of men and 37.8% of women had a MAR below 0.75 for 10 nutrients, indicating an insufficiency in nutrient intake. When the Index of INQ falls below 1, it signifies that specific nutrient intake is inadequate in relation to energy intake, implying that energy intake needs to be excessive to meet the RDA for those specific nutrients. In terms of vitamins, both fat-soluble vitamin A and water-soluble vitamin C were notably below the EAR. As previously mentioned, the insufficient consumption of vitamins A and C among internship students can likely be attributed to a comparatively lower intake of fruits and vegetables. Transitioning to minerals, the calcium intake of the interns proved insufficient, falling short of the EAR, while their consumption of potassium did not attain the AI. Notably, calcium displayed the lowest NAR and INQ scores among the minerals. This lower consumption of calcium may be linked to their reduced consumption of dairy products while on the ship.

### Contribution of food groups to daily nutrients intake

As a result of investigating the main source of dietary energy and nutrients, different results were confirmed according to gender. Among men students, their primary sources of energy and carbohydrates were grains and potatoes, followed by meat, fish, and eggs, and vegetables and fruits contributing to their calcium intake. Interestingly, a similar pattern was noted for women students, where grains and potatoes were the main energy and carbohydrate sources, but strikingly, their intake of meat, fish, and eggs also played a significant role as energy and carbohydrate sources. Notably, vegetables and fruits were the primary contributors to calcium intake for female internship participants. These findings contrast with the conventional understanding of typical nutrient sources, particularly carbohydrates and calcium [15–17]. These disparities highlight dietary issues such as an imbalance between dietary energy and physical activity, inadequate consumption of plant-based foods including vegetables, fruits, grains, and potatoes, insufficient dairy product intake, and elevated consumption of animal-based foods rich in cholesterol and fat.

### Factors contributing to insufficient nutrient intake among shipboard internships students

Insufficient nutrient intake among internship students is believed to arise from a combination of factors, encompassing both human resources and facility-related aspects [29, 30]. Typically, there is no dietitian present onboard, and food selection and preparation are overseen by a cook [3]. As the qualifications for selecting cooks do not encompass detailed nutritional knowledge, it is conceivable that various nutritional deficiencies, such as those identified in this cross-sectional study, may arise. Furthermore, given that the majority of meals are self-serve, internship students have the freedom to select suitable food options from the provided menu. However, it is apparent that even boarding students lacked the necessary nutritional knowledge, leading to suboptimal nutrient intake. Moreover, we may consider the infrastructural limitations of ships for storage and cooking [3]. As we observed in our data, internship students have less tendency to take fruits and vegetables, and these food sources are closely intertwined with storage and preservation technologies. The edibility and nutritional quality of fruits and vegetables are contingent upon variables such as temperature, moisture, and other factors that influence the proliferation of spoilage-causing organisms. Due to the nature of long-term sea voyages, fruits and vegetables should be stored with traditional (drying, salting, and smoking) and modern storage techniques (canning and compressed-gas refrigeration) [31]. Progress in food storage techniques can enhance the preservation of fruits and vegetables, yet there is a threshold for maintaining their freshness over extended periods. As a result, there could be constraints on how much internship students can enjoy fruits and vegetables. The limitation of cooking firepower can significantly impact food choices and cooking methods [4, 5]. Due to the restriction on acquiring fresh food for internship participants, various cooking methods using fire have become the primary means of preparing dishes. There are numerous cooking techniques involving fire, such as baking, frying, roasting, grilling, steaming, simmering, broiling, blanching, braizing, and stewing. However, the ship provides a confined and compact space, resulting in limited cooking firepower to mitigate potential safety issues [3]. A lower cooking firepower can greatly compromise the sensory quality of the dishes provided to internship participants. Therefore, a future follow-up comparative study could be conducted to investigate how the participants make food selections and choices outside of the ship.

### Limitations

Our study has a significant limitation in interpreting the dietary patterns consumed by internship participants due to our utilization of a cross-sectional design with a small sample size. Consequently, understanding the causality of eating patterns by students and gathering information on dynamic longitudinal effects is challenging. However, despite the limitations of the study design, our research provides valuable insights into the potentially improper selection of dietary patterns for internship participants who are future seafarers, utilizing an in-depth 24-hour dietary recall test. While the conventional 24-hour dietary recall method offers advantages in terms of remote and convenient accessibility, it also has drawbacks, as answers reliant on memory could introduce biases to nutritional data. However, in our study, internship participants submitted photographs taken before and after meals. Consequently, the data obtained from the 24-hour dietary recall method underwent verification by experienced dietitians.

## Conclusion

After it was the discovery that scurvy among seafarers as being caused by vitamin C deficiency, despite revolutionary technological advancements, contemporary seafarers still face a variety of nutritional deficiencies, including vitamin C, as evidenced by our observations. These deficiencies can be attributed to factors such as limited access to fresh foods, confinement to ships, and a lack of diverse dietary options. Consequently, there is a need to prioritize the maintenance of seafarers' health by providing additional nutritional education tailored to their circumstances. Furthermore, it is worth considering the implementation of remote nutritional consulting services delivered by registered dietitians. This approach could offer seafarers expert guidance on maintaining optimal nutrition despite the challenges posed by their unique environment.

## Data Availability

The data used in this study are not available to the public due to ethical restrictions. However, with reasonable request and subject to written agreement from all study sites and study authors, deidentifed data used in this study may be made available from corresponding author.

## Abbreviations

AI: Adequate intake
AMDRs: Acceptable macronutrient distribution ranges
CDRR: Chronic disease risk reduction intake
EAR: Estimated average requirement
EER: Estimated energy requirement
INQ: Index of nutritional quality
KDRIs: Dietary Reference Intakes for Koreans
NAR: Nutrient adequacy ratio
MAR: Mean adequacy ratio
